# Involvement of Insulin Signaling Disturbances in Bisphenol A-Induced Alzheimer’s Disease-like Neurotoxicity

**DOI:** 10.1038/s41598-017-07544-7

**Published:** 2017-08-08

**Authors:** Tingwei Wang, Cuiwei Xie, Pengfei Yu, Fangfang Fang, Jingying Zhu, Jie Cheng, Aihua Gu, Jun Wang, Hang Xiao

**Affiliations:** 1 Department of Toxicology, the Key Lab of Modern Toxicology (NJMU), Ministry of Education, School of Public Health, Nanjing Medical University, Nanjing, 211166 China; 2 Wuxi Center for Disease Control and Prevention, 499 Jincheng Road, Liangxi District, Wuxi, Jiangsu 214023 China

## Abstract

Bisphenol A (BPA), a member of the environmental endocrine disruptors (EDCs), has recently received increased attention because of its effects on brain insulin resistance. Available data have indicated that brain insulin resistance may contribute to neurodegenerative diseases. However, the associated mechanisms that underlie BPA-induced brain-related outcomes remain largely unknown. In the present study, we identified significant insulin signaling disturbances in the SH-SY5Y cell line that were mediated by BPA, including the inhibition of physiological p-IR Tyr1355 tyrosine, p-IRS1 tyrosine 896, p-AKT serine 473 and p-GSK3α/β serine 21/9 phosphorylation, as well as the enhancement of IRS1 Ser307 phosphorylation; these effects were clearly attenuated by insulin and rosiglitazone. Intriguingly, Alzheimer’s disease (AD)-associated pathological proteins, such as BACE-1, APP, β-CTF, α-CTF, Aβ _1–42_ and phosphorylated tau proteins (S199, S396, T205, S214 and S404), were substantially increased after BPA exposure, and these effects were abrogated by insulin and rosiglitazone treatment; these findings underscore the specific roles of insulin signaling in BPA-mediated AD-like neurotoxicity. Thus, an understanding of the regulation of insulin signaling may provide novel insights into potential therapeutic targets for BPA-mediated AD-like neurotoxicity.

## Introduction

Bisphenol A (BPA), a member of the environmental endocrine-disrupting chemicals (EDCs), is widely used in carbonated beverages and polyester food packing material, tooth solid sealing agents, baby bottles, infusion bags and other products with additives^1^. Widespread and continuous exposure to BPA in humans has been confirmed by biomonitoring studies in general populations, and individuals are at risk from internal exposure to unconjugated BPA^2, 3^. According to a recent National Health and Nutrition Examination Survey, nearly all US citizens exhibit detectable amounts of BPA metabolites in urine and blood^4, 5^.

Insulin is released from the pancreas into the bloodstream and can cross the blood-brain barrier (BBB) via a carrier-facilitated process; it is also secreted by the hippocampus, the prefrontal cortex and other regions in the brain^4^. Numerous studies have indicated that insulin binds to its receptors and plays an important role in the maintenance of brain neuronal survival, energy metabolism homeostasis, learning and memory^5^.

The insulin receptor (IR) is densely expressed in pyramidal cell axons in the hippocampal CAl region and is mainly distributed in the dominant learning, memory and cognitive function regions of the brain^6^. Under physiological conditions, insulin signaling is mediated by the IR tyrosine kinase receptor family. When insulin binds to its receptor, the IR tyrosine kinase is activated, which induces intracellular insulin receptor substrate (IRS) protein tyrosine phosphorylation^7^ and subsequently results in the activation of phosphatidylinositol 3-kinase (PI3K) and serine/threonine protein kinase B (protein kinase B, AKT)^8^, which have been shown to be involved in the regulation of insulin metabolism^9^.

BPA exposure has recently been demonstrated to be a risk factor for insulin resistance and metabolic disorders^10^. Our previous findings indicated that perinatal BPA exposure contributed to peripheral insulin resistance in offspring during adulthood^11^. In parallel with the peripheral insulin resistance induced by BPA, intriguingly, perinatal BPA exposure also resulted in brain insulin resistance in offspring when they were 8 months of age^12^. Adult male mice treated with a subcutaneous injection of 100 μg/kg/d BPA for 30 days exhibited a significant decrease in insulin sensitivity and glucose transporter 1, 3 (GLUT1, 3) protein levels in the brain^13^. These findings support disturbances in insulin signaling induced by BPA in both peripheral and central systems.

AD is characterized by the deposition of two types of filamentous aggregates: the formation of senile plaques (SP) from amyloid-β (Aβ) and neurofibrillary tangles composed of phosphorylated tau^14^. Amyloid precursor protein (APP) forms Aβ polypeptide through a series of proteolytic reactions and exerts neurotoxic effects. These hydrolyzed polypeptides aggregate to form Aβ and amyloid protein and ultimately form SP.

The association between insulin resistance and Alzheimer’s disease (AD) has recently received substantial attention. Individuals with type 2 diabetes have approximately double the chances of developing AD^15^. Hoscheidt *et al*. have suggested that an increased HOMA-IR was associated with increased sAβPPβ and Aβ_42_^16^. Moreover, impaired glucose metabolism in the brains of individuals with AD is a widely recognized early feature of the disease^17^. Considering the aforementioned studies and our previous work, we hypothesized that insulin signaling disturbances induced by BPA might induce APP and p-tau enhancement, thereby contributing to AD-like neurotoxicity. Considering the environmental estrogenic activity of BPA, the roles of estrogen receptors (ERs; ER-α, ER-β and GPR30) in the regulation of insulin signaling were also discussed.

## Results

### Effects of BPA on the expression of insulin signaling pathway components in SH-SY5Y cells

**Figure 1:**
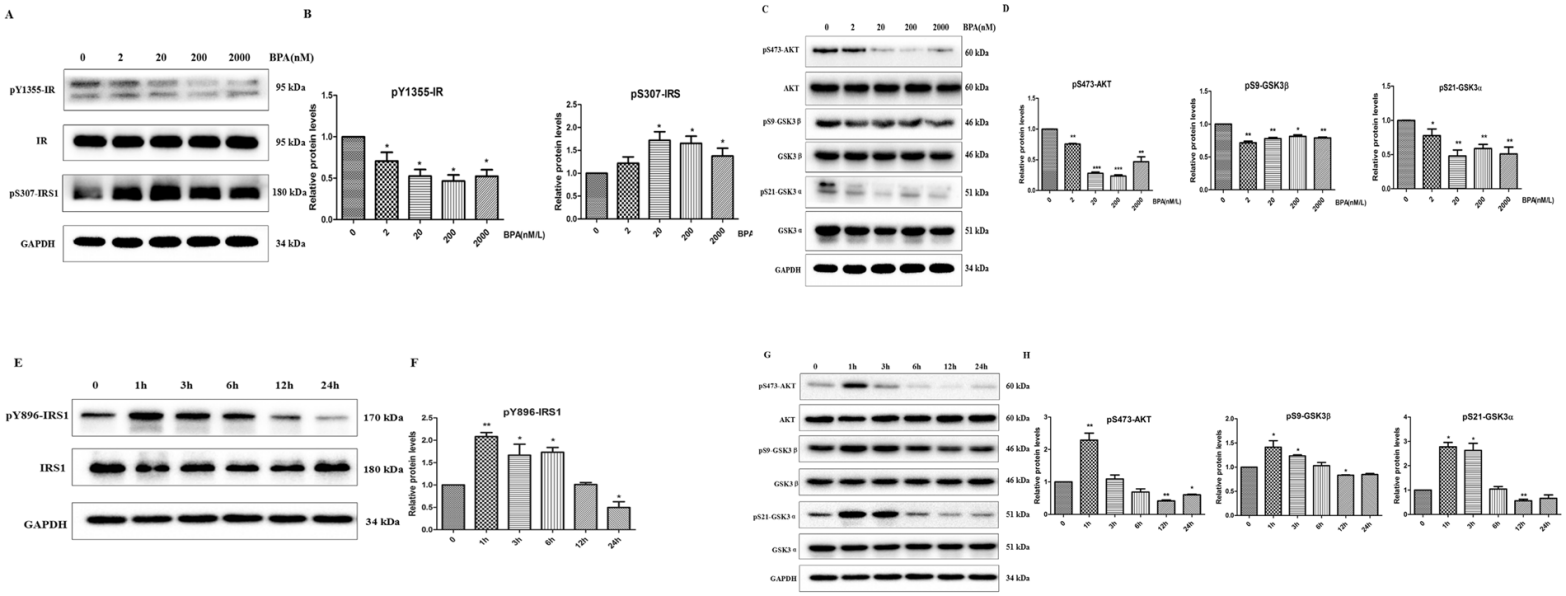
BPA disturbed the insulin signaling pathway. Western blot expression analysis of (**A**) different concentrations of BPA (0, 2, 20, 200, and 2000 nM/L) on IR phosphorylation and IRS phosphorylation in SY5Y cells. (**C**) Effect of different concentrations of BPA (0, 2, 20, 200, and 2000 nM/L) on AKT and GSK3α/3β phosphorylation in SY5Y cells. (**E**) IR phosphorylation and IRS phosphorylation in SY5Y cells at various time points (0, 1, 3, 6, 12, and 24 h) of 20 nM/L BPA treatment. (**G**) AKT, AKT phosphorylation, GSK3α/3β, and GSK3α/3β phosphorylation in SY5Y cells at various time points (0, 1, 3, 6, 12, and 24 h) of 20 nM/L BPA treatment. (**B**,**D**,**F**,**H**) GAPDH levels detected in parallel served as controls. Mean values ± SEMs are representative of three independent isolations and three independent samples. Significant differences between the treatment groups and the control group were determined via one-way ANOVA and the Dunnett multiple comparison procedure. (*P < 0.05, **P < 0.01, ***P < 0.001 compared with the control group).

The IR and IRS-1 play critical roles in insulin signaling activation and transduction^18^; thus, we investigated the phosphorylation of the IR and IRS-1. SH-SY5Y (SY5Y) cells were incubated with different concentrations of BPA (0, 2, 20, 200, or 2000 nM) for 12 hours; the phosphorylation site of the insulin receptor (IR) p-IR Tyr1355 was subsequently detected via western blotting. As indicated in Fig. 1A, BPA exposure significantly decreased the p-IR Tyr1355 level; moreover, it substantially enhanced the expression of IRS1 Ser307 (a phosphorylation site that inhibits insulin signaling by antagonizing tyrosine phosphorylation), a key downstream signaling component of the IR, with the maximal level at the concentration of 20 nM (P < 0.05). As a downstream signaling mechanism of IRS-1, the alteration of Kinase B/AKT (AKT) activity is one key characteristic of insulin resistance. Figure 1C indicates a striking reduction in the phosphorylation level of AKT Ser473 after treatment with various concentrations of BPA. In addition, the expression of pS9-GSK3β, a protein with a phosphorylation site that is key to the activity of GSK3β, was significantly decreased (P < 0.05). Similar results were also identified for pS21-GSK3α. Twenty nM BPA significantly affected all insulin signaling components assessed; thus, this dose was selected for the evaluation of BPA-induced insulin signaling disturbances at various time points (0, 1, 3, 6, 12, and 24 h). Figure 1E indicates that BPA exposure clearly increased the expression of pY896-IRS1 at 1, 3 and 6 h; the expression then gradually decreased, with the maximal decrement occurring at 24 h. In addition, the AKT Ser473 phosphorylation was decreased from 6 h. Consistent with the expression of AKT Ser473 phosphorylation, the pS9-GSK3β and pS21-GSK3α phosphorylation was also substantially decreased, with the peak effect at 12 h (Fig. 1G). To further confirm the involvement of the insulin signaling pathways in BPA-induced neural insulin resistance, mTOR and PP2Ac, which located downstream of AKT, were explored in the present work. As depicted in Fig. S1, BPA exposure significantly reduced the phosphorylation of mTOR and the methylation level of PP2Ac, both of which were linked to p-tau phosphorylation^19, 20^.

### BPA exposure contributes to intracellular [Ca^2+^]_i_ and ROS increase, and ATP and mitochondrial membrane potential decrease in SY5Y cells

Since mitochondrion associated dysfunctions, including calcium abnormalities, ROS generation, ATP reduction and mitochondrial membrane potential decrease can be directly linked to the altered tau phosphorylation and amyloid precursor protein processing that are defining features of AD^21–23^, we then investigated whether BPA exposure affected the associated dysfunctions of mitochondrion aforementioned. As shown in Fig. S2B, BPA treatment triggered a transient Ca^2+^ increase, then returned to baseline, while in control group (DMSO lower than 0.5%), no obvious Ca^2+^ increase was observed. Moreover, with the laser confocal assay, it was indicated that BPA treatment markedly increased the level of ROS (Fig. S2D). Meanwhile, ATP generation was significantly decreased as well as mitochondrial membrane potential after BPA exposure (Fig. S2A and C), suggesting the potential roles of BPA in the formation of AD like pathological changes.

### Effects of BPA on the expressions of APP, BACE-1 and Aβ_1–42_ proteins

**Figure 2:**
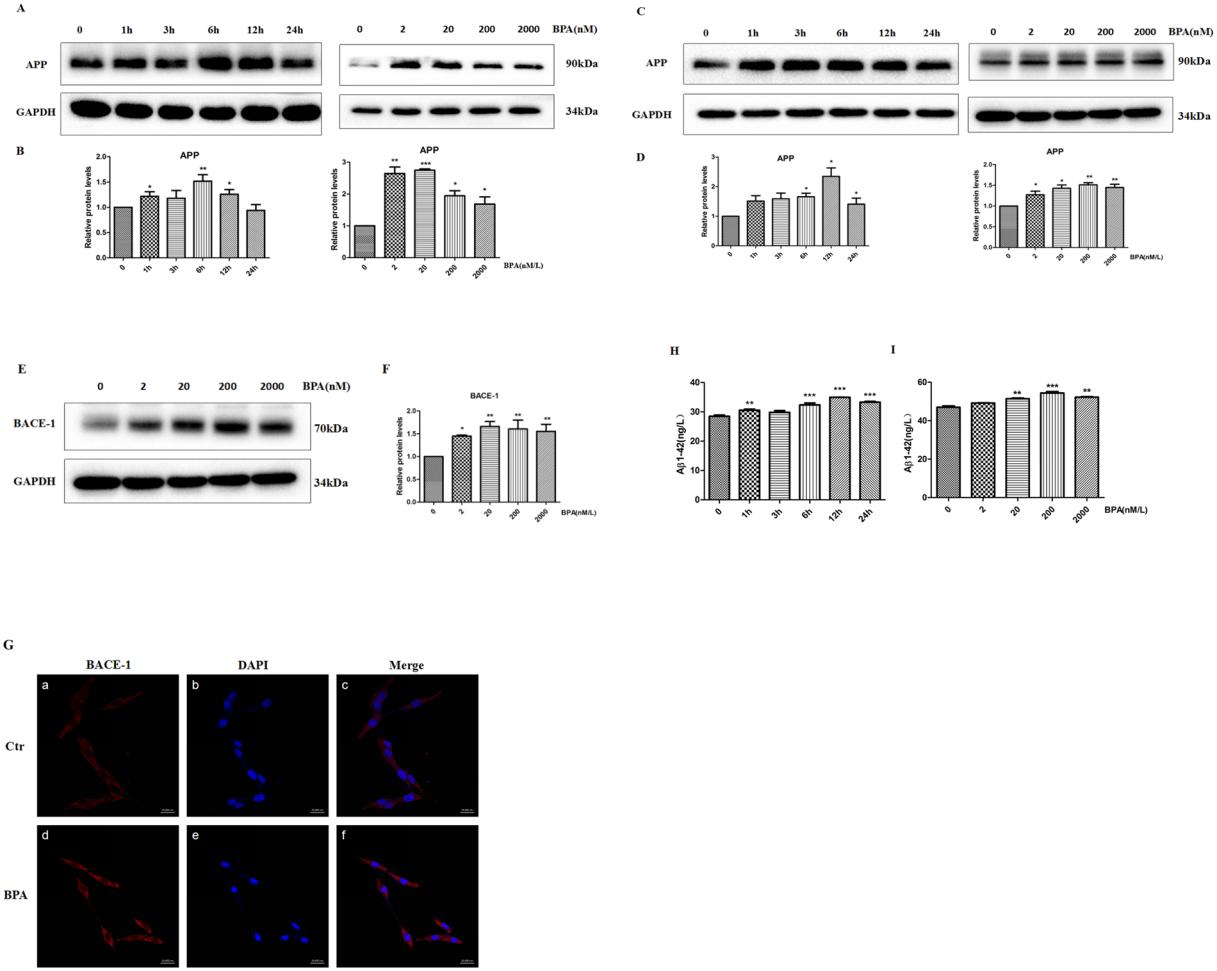
BPA upregulated APP, BACE-1 and Aβ_1–42_ expressions. Western blot analysis of the expression of (**A**) APP protein in SY5Y cells treated with different concentrations of BPA (0, 2, 20, 200, and 2000 nM/L) at various time points (0, 1, 3, 6, 12 and 24 h). (**C**) APP and BACE-1 protein expressions in PC-12 cells at different concentrations of BPA (0, 2, 20, 200, and 2000 nM/L) at various time points (0, 1, 3, 6, 12 and 24 h). (**B**,**D**,**F**) GAPDH levels were assessed in parallel and served as controls. Data are presented as the mean ± SEM from three independent experiments. (**E**) Effect of different concentrations of BPA (0, 2, 20, 200, and 2000 nM/L) on BACE-1 expression in PC-12 cells. (**G**) Immunofluorescence analysis of the expression of BACE-1 protein after treatment with 20 nM/L BPA. (**H**,**I**) Effects of different concentrations of BPA on Aβ_1–42_ expression in PC-12 cells determined via ELISA. Mean values ± SEMs are representative of three independent isolations and three independent samples. Significant differences between the treatment groups and the control group were determined via one-way ANOVA and the Dunnett multiple comparison procedure. (*P < 0.05, **P < 0.01, ***P < 0.001 compared with the control group).

Brain insulin signaling disturbances are closely associated with AD pathology^16^; thus, we subsequently investigated whether the BPA-induced disturbances of insulin signaling resulted in pathological molecular up-regulation. SY5Y cells were incubated with various doses of BPA (0, 2, 20, 200, or 2000 nM) for 12 h, and the expression of the pathological protein APP (Aβ_1–42_ precursor) was detected. As indicated in Fig. 2A, BPA substantially enhanced the expression of APP at a concentration of 2 nM, and the effect further increased at 20, 200 and 2000 nM. With 20 nM BPA treatment, the APP expression rapidly increased to a peak at 12 h and then gradually decreased to a steady level. To further confirm the specific effects of BPA on the upregulation of APP, PC-12, another cell line that is commonly used for neural degenerative diseases, was applied to validate the toxic effects mediated by BPA. As shown in PC-12 cells, BPA treatment significantly enhanced the APP expression at various concentrations and time points (Fig. 2C and D), which verifies the specific roles of BPA in APP regulation. Pathophysiologically, increased APP expression, together with altered proteolysis, results in the accumulation of Aβ peptides that can aggregate. Therefore, an ELISA assay was performed to detect the secretion of the downstream pathological protein Aβ_1–42_. As indicated in Fig. 2H and I, the Aβ_1–42_ secretion mediated by BPA was significantly increased at the doses of 20, 200 and 2000 nM, and exposure to 20 nM BPA substantially enhanced the Aβ_1–42_ secretion from 6 to 24 h; these findings suggest that BPA, even at a low dose, promoted Aβ_1–42_ generation. Furthermore, we evaluated the expression of BACE-1, one of the key enzymes responsible for APP proteolysis, which promotes Aβ_1–42_ generation^24^. Immunofluorescent staining of the BACE-1 expression was performed in PC-12 cells cultured with BPA. The fluorescence labeling for BACE-1 was strikingly enhanced by BPA treatment (Fig. 2G), and the upregulation of BACE-1 was further corroborated by a western blot assay, which indicated that BPA treatment substantially elevated the expression of the BACE-1 fragment (Fig. 2E). To further confirm the specific effects of BPA on APP proteolysis, the retention of corresponding membrane-anchored C-terminal fragments of APP were detected, as shown in Fig. S3, BPA exposure obviously increased the β-CTF and the α-CTF, demonstrating the activation of BACE-1 by BPA. Furthermore, BPA mediated APP proteolysis can be partially attenuated by rosiglitazone, implying the protective effects of insulin signaling pathways in BPA-mediated APP proteolysis.

### Effects of BPA on phosphorylated tau in SH-SY5Y and PC-12 cells

**Figure 3:**
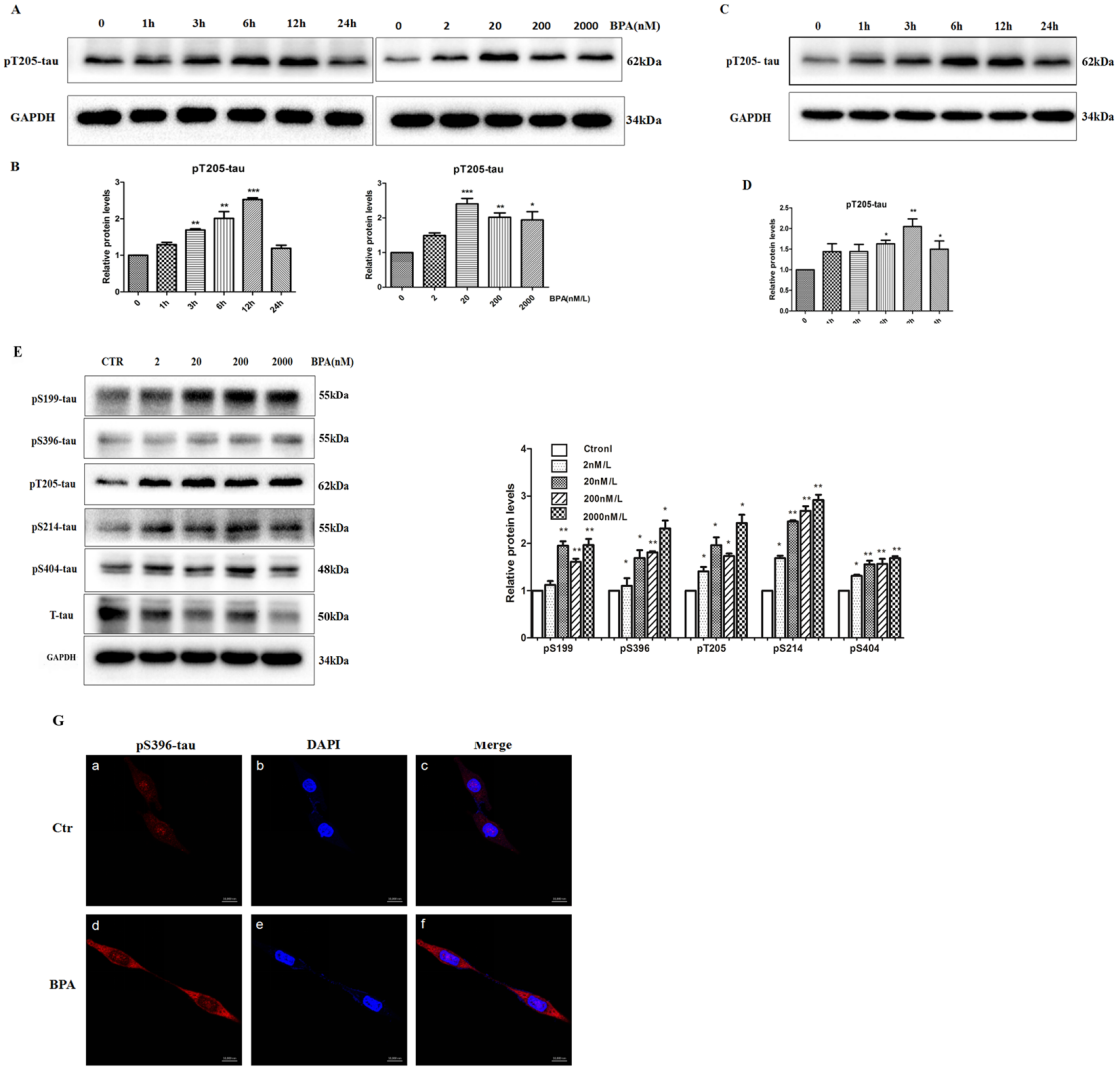
BPA enhanced the expression of phosphorylated tau. Western blot analysis indicated the expression of phosphorylated tau in SY5Y cells treated with 20 nM/L BPA (**A**) at different concentrations (0, 2, 20, 200, and 2000 nM/L) and various time points (0, 1, 3, 6, 12, and 24 h). (**C**) Expression of phosphorylated tau in PC-12 cells treated with 20 nM/L BPA at various time points (0, 1, 3, 6, 12, and 24 h) of BPA. (**E**) Expression of phosphorylated tau in PC-12 cells treated with different concentrations of BPA (0, 2, 20, 200, and 2000 nM/L). (**D**,**F**) GAPDH levels were detected in parallel and served as controls. (**G**) Immunofluorescence analysis of the expression of p-tau after treatment with 20 nM/L BPA. Mean values ± SEMs are representative of three independent isolations and three independent samples. Significant differences between the treatment groups and the control group were determined via one-way ANOVA and the Dunnett multiple comparison procedure. (*P < 0.05, **P < 0.01, ***P < 0.001 compared with the control group).

Enhanced tau phosphorylation is another key feature during the development of AD^25^; thus, we subsequently aimed to investigate whether BPA exposure contributed to the enhancement of phosphorylated tau (p-tau). As indicated in Fig. 3A, treatment with BPA (0, 2, 20, 200, or 2000 nM) in SY5Y cells resulted in a significant increase in the p-tau expression at the site of Tyr205, and the maximal dose response to BPA treatment was 20 nM. Using this dose, SY5Y cells were incubated with BPA for various durations (from 0 to 24 h); the pT205-tau expression was substantially increased from 3 to 24 h, with a peak response at 12 h, and the expression subsequently decreased to a steady level. To further verify the toxic effects of BPA on neural cells, another cell line, PC-12, was used in the current work. The results of the western blot assay presented in Fig. 3E indicate that BPA exposure resulted in the activation of several phosphorylated sites of the tau protein, including Ser199, Ser396, Thr205, Ser214 and Ser404. In addition, the immunofluorescence technique was used to investigate the effects of BPA on p-tau in PC-12 cells. As shown in Fig. 3G and H, the fluorescence of pSer396-tau was substantially increased in the cytoplasm compared with the control group, which emphasizes the roles of BPA in this process. To confirm the aggregation of the pathological proteins mediated by BPA, the thioflavine-S staining assay was used in the current work, as depicted in Fig. S4, BPA treatment obviously increased the fluorescence density when compared with the control group, suggesting the effects of BPA on the pathological protein aggregation.

### Effects of ERs on BPA-mediated pathological protein expression

**Figure 4:**
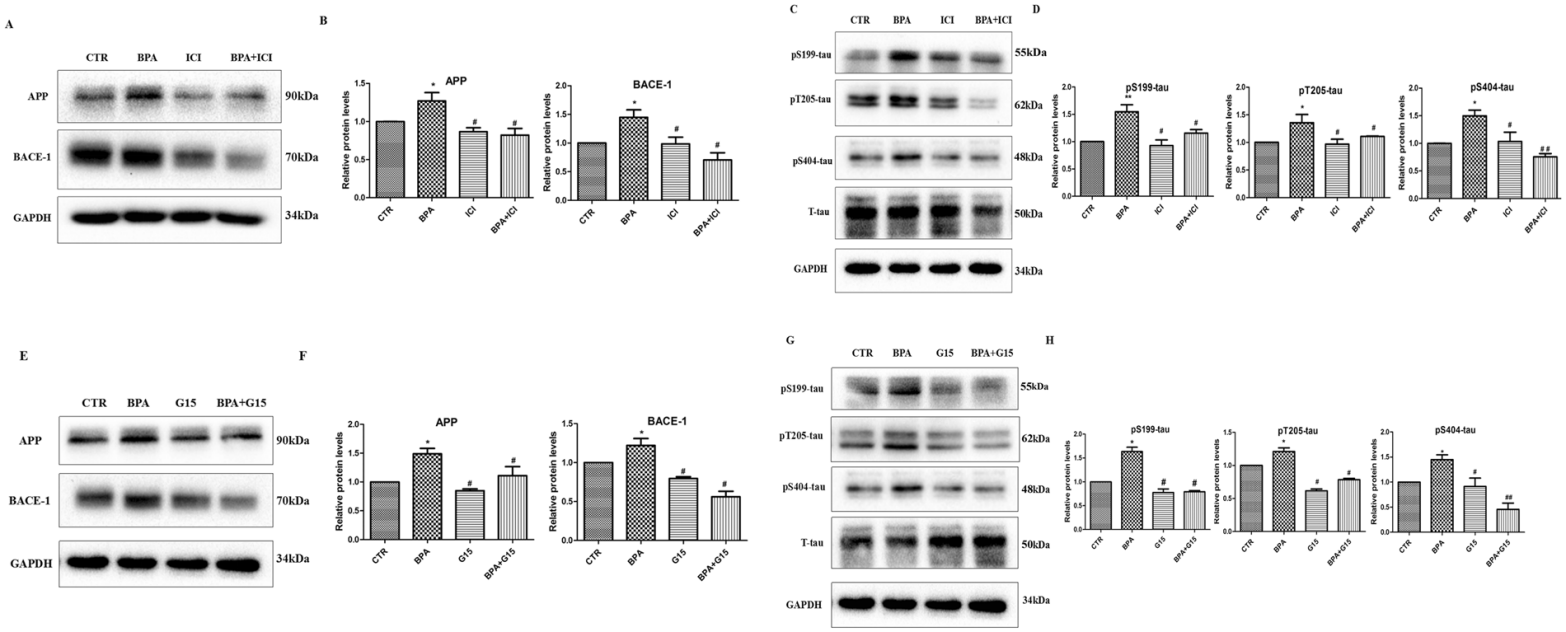
Estrogen receptors were involved in BPA-induced APP, BACE-1 and p-tau expressions. (**A**) ICI182780 inhibited the BPA-induced expressions of APP and BACE-1 in PC-12 cells. (**C**) ICI182780 inhibited the BPA-induced expression of p-tau in PC-12 cells. (**E**) G15 inhibited the BPA-induced expressions of APP and BACE-1 in PC-12 cells. (**G**) G15 inhibited the BPA-induced expression of p-tau in PC-12 cells. (**B**,**D**,**F**,**H**) GAPDH levels were assessed in parallel and served as controls. Mean values ± SEMs are representative of three independent isolations and three independent samples. Significant differences between the treatment groups and the control group were determined via one-way ANOVA and the LSD Dunnett comparison procedure. (*P < 0.05, **P < 0.01, ***P < 0.001 compared with the control group).

BPA binds to ERs and exerts estrogen-like effects^26^; thus, it was reasonable that ERs may be involved in BPA-induced pathological effects. PC-12 cells were pre-incubated with the estrogen receptor ERα/β inhibitor ICI 182780 (30 μM) or the GPR30 inhibitor G15 (30 μM) for 30 min prior to BPA treatment,since both of 30 μM ICI 182780 and G15 did not obviously affect cell viability (Fig. S5). The western blot assay results indicated that ICI 182780 substantially decreased the BPA-induced APP, BACE-1 (Fig. 4A) and p-tau protein expressions in the Ser199, Thr205, and Ser404 phosphorylation sites (Fig. 4C and D). Moreover, G15, an antagonist of GPR30, was demonstrated to ameliorate APP, BACE-1 (Fig. 4E and F), and p-tau Thr205, Ser404 and Ser199 expressions (Fig. 4G and H). Thus, these findings emphasize the potential roles of ERs in BPA-induced pathological protein regulation.

### Specific effects of BPA on insulin signaling

**Figure 5:**
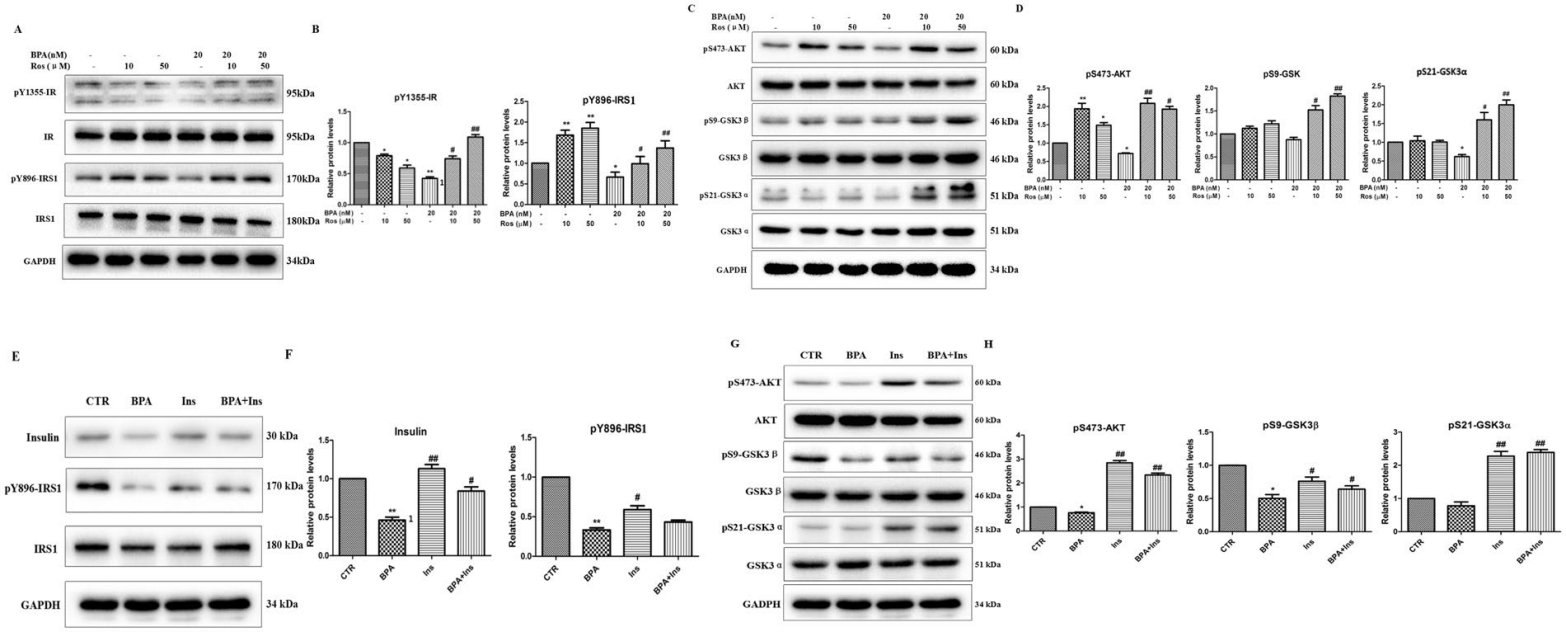
Effects of rosiglitazone and insulin on BPA-induced insulin signaling pathways. (**A**) Effects of rosiglitazone on the expression of IR and IRS tyrosine phosphorylation mediated by BPA in SY5Y cells. (**C**) Effects of rosiglitazone on the expression of AKT and GSK3α/3β serine phosphorylation mediated by BPA in SY5Y cells. (**E**) Effects of insulin on the expression of phosphorylated IR and phosphorylated IRS mediated by BPA in SY5Y cells. (**G**) Effects of insulin on the expression of phosphorylated AKT and GSK3α/3β mediated by BPA in SY5Y cells. (**B**,**D**,**F**,**H**) GAPDH levels were assessed in parallel and served as controls. Mean values ± SEMs are representative of three independent isolations and three independent samples. Significant differences between the treatment groups and the control group were determined via one-way ANOVA and the Dunnett multiple comparison procedure. (*P < 0.05, **P < 0.01 compared with the BPA single treatment group, ^#^P < 0.05, ^##^P < 0.01).

Having determined that BPA exposure resulted in the disturbance of insulin signaling, rosiglitazone and insulin, which activate and enhance insulin signaling transduction, were used in the present work. SY5Y cells were co-incubated with BPA (20 nM) and rosiglitazone (10 μM, 50 μM) or insulin (200 nM) for 12 h, and the insulin signaling pathways were investigated via western blotting. As indicated in Fig. 5A, exposure to 20 nM BPA substantially decreased the expression of pY1355-IR and the key downstream signaling molecule pY896-IRS-1, and these effects were partially rescued by rosiglitazone. To further confirm the function of rosiglitazone in insulin signaling, another important signaling molecule, pS473-AKT, was also detected. As indicated in Fig. 5C, rosiglitazone treatment substantially restored the BPA-induced down-regulation of pS473-AKT, and similar results were obtained for the insulin treated group (Fig. 5E and G), which demonstrated the specific effects of BPA on insulin signaling. Moreover, both rosiglitazone and insulin significantly decreased BPA-induced GSK3α and GSK3β activation, as evidenced by the increased phosphorylation of pS21-GSK3α and pS9-GSK3β. These findings support the specific effects of BPA on insulin signaling.

### Involvement of insulin signaling in BPA-induced APP, BACE-1 and Aβ_1–42_ expression

**Figure 6:**
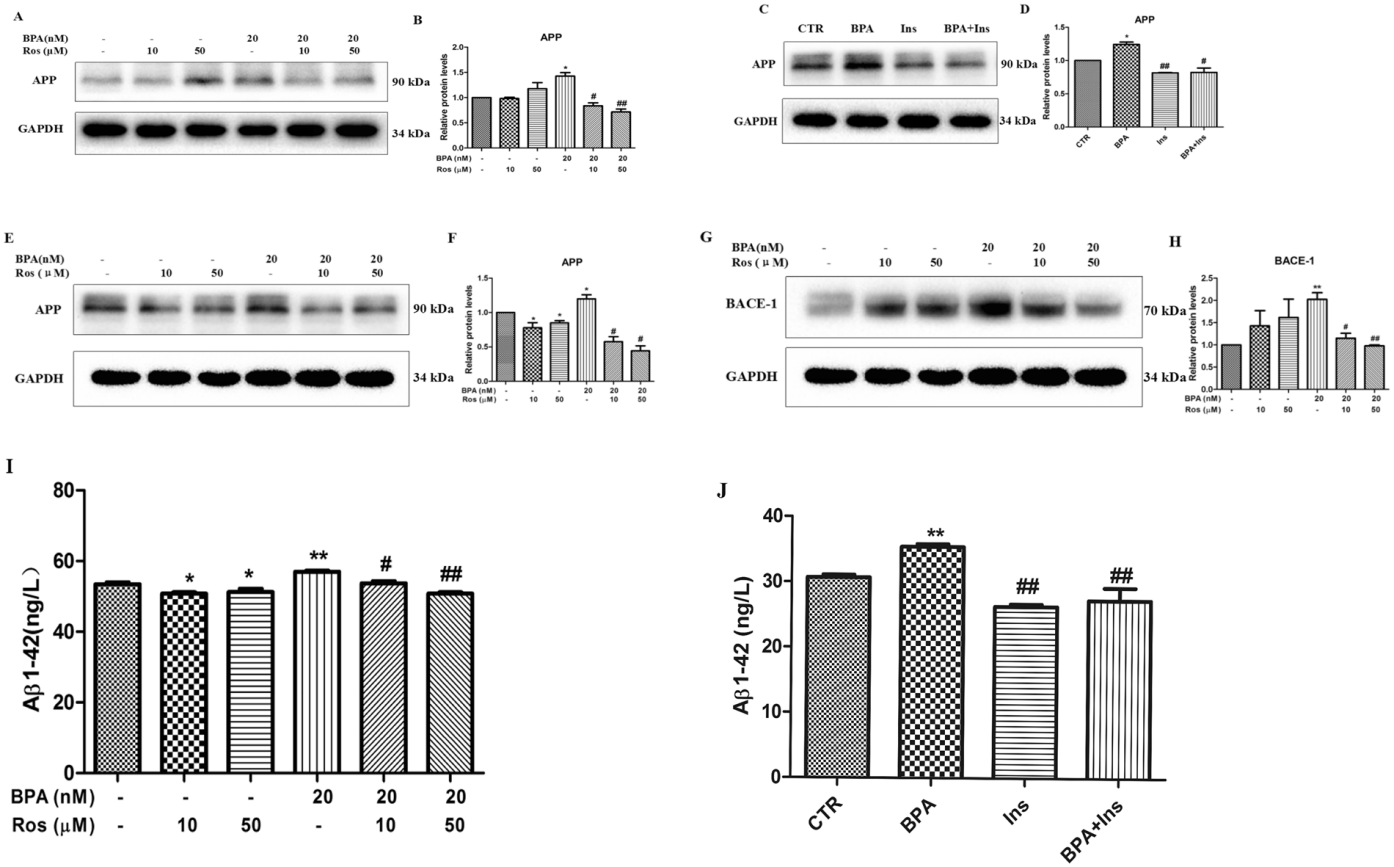
Effects of rosiglitazone on BPA-induced APP, BACE-1 and Aβ_1–42_ expressions. (**A**) Effect of rosiglitazone on the expression of APP protein mediated by BPA in SY5Y cells. (**C**) Effect of rosiglitazone on the expression of APP protein mediated by BPA in PC-12 cells. (**E**) Effect of rosiglitazone on the expression of BACE-1 protein mediated by BPA in PC-12 cells. (**B**,**D**,**F**,**H**) GAPDH levels were assessed in parallel and served as controls. (**I**) ELISA analysis of the effects of rosiglitazone on Aβ_1–42_ expression in PC-12 cells. (**J**) ELISA analysis of the effects of insulin on Aβ_1–42_ expression in PC-12 cells. Mean values ± SEMs are representative of three independent isolations and three independent samples. Significant differences between the treatment groups and the control group were determined via one-way ANOVA and the Dunnett multiple comparison procedure. (*P < 0.05, **P < 0.01, ***P < 0.001 compared with the BPA single treatment group, ^#^P < 0.05, ^##^P < 0.01).

BPA clearly hampered insulin signal transduction and increased the APP, BACE-1 and Aβ_1–42_ expressions; thus, the next logical step was to dissect the roles of insulin signaling in APP, BACE-1 and Aβ_1–42_ expressions. Figure 6A and C indicates that when SY5Y cells were co-incubated with rosiglitazone and/or insulin, the BPA-induced up-regulation of APP was substantially decreased. In parallel with data in SY5Y cells, a similar result was obtained in PC-12 cells when rosiglitazone was applied (Fig. 6E). Moreover, rosiglitazone treatment clearly mitigated the BPA-induced upregulation of BACE-1 (Fig. 6G) and suppressed Aβ_1–42_ excretion (Fig. 6I), which demonstrated the involvement of insulin signaling in BPA-induced pathological protein regulation.

### Involvement of insulin signaling in BPA-induced hyperpho**s**phorylation of p-tau

**Figure 7:**
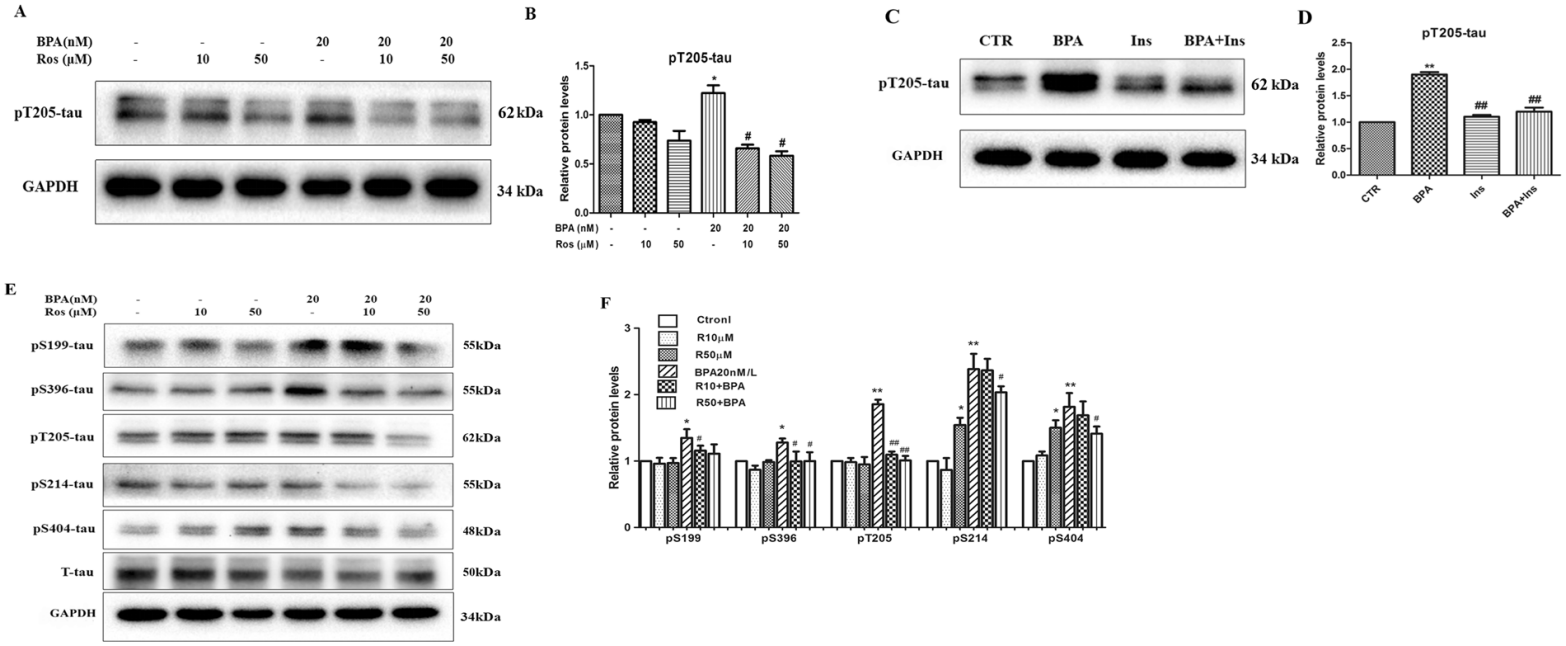
Effects of rosiglitazone and insulin on BPA-induced tau phosphorylation. (**A**) Effect of rosiglitazone on the expression of phosphorylated tau mediated by BPA in SY5Y cells. (**C**) Effect of insulin on the expression of phosphorylated tau mediated by BPA in SY5Y cells. (**E**) Effect of rosiglitazone on the expression of phosphorylated tau mediated by BPA in PC-12 cells. (**B**,**D**,**F**) GAPDH levels were assessed in parallel and served as controls. Mean values ± SEMs are representative of three independent isolations and three independent samples. Significant differences between the treatment groups and the control group were determined via one-way ANOVA and the Dunnett multiple comparison procedure. (*P < 0.05, **P < 0.01 compared with the BPA single treatment group, ^#^P < 0.05, ^##^P < 0.01).

We also assessed whether insulin signaling was implicated in the BPA-induced hyperphosphorylation of p-tau. SY5Y cells were co-incubated with BPA and rosiglitazone or insulin; the p-tau expression was subsequently examined. BPA induced a striking increase in the expression of pT205-tau, pS199-tau, pS396-tau and pS214-tau, and these effects were significantly ameliorated in cells treated with rosiglitazone and/or insulin (Fig. 7A and C, Fig. S6), which suggests the roles of insulin signaling in this process. To further corroborate this finding, the experiments were also conducted in PC-12 cells; as speculated, rosiglitazone substantially attenuated the BPA-induced hyperphosphorylation of p-tau at differential sites, including pS199, pS396, pT205, pS214 and pS404 (Fig. 7E). These findings strengthen the hypothesis that insulin signaling participates in the BPA mediated hyperphosphorylation of tau protein.

## Discussion

Earlier studies on BPA primarily focused on reproductive and developmental toxicity^27^, and recent studies have identified diverse pathological effects attributable to exposure to even a low dose of BPA. Epidemiological reports and animal experiments have indicated a correlation between BPA exposure and cognitive and behavioral alterations^28^. Based on these phenomena, numerous studies have begun to address the cellular and molecular mechanisms that underlie BPA-mediated brain damage, such as mitochondrial dysfunction, neuroinflammation and increased reactive oxygen species production^29^. To identify and characterize additional molecular pathways that might be involved in BPA-induced deleterious actions, we extended our studies of BPA-induced neural toxicity by further investigating the effects of insulin signaling in this process.

Brain insulin maintains the balance of neuronal energy, regulates neural cell proliferation, differentiation, neurotransmitter release^30^, and axon growth, and prevents oxidative stress^31^. It is released from the pancreas into the bloodstream and can cross the BBB and enter the brain. It can also be secreted by the hippocampus, frontal lobe and other brain regions^32^. Our previous studies have indicated that BPA exposure significantly affected insulin secretion and sensitivity, which contributed to peripheral insulin resistance in both offspring exposed perinatally to BPA and adult mice^11^. Intriguingly, we recently extended our research and determined that BPA exposure resulted in brain insulin resistance in mice, which demonstrates the disturbance in the insulin signaling pathways^12^.

The IR is highly expressed in the brain^33^. IR activation in the brain is proposed to be responsible for insulin-induced enhancement of cognitive function in human subjects and rodents^32^. In the brain, insulin binds to the IR, which activates the tyrosine kinase domain of the β-subunits, thereby leading to autophosphorylation. The phosphorylation of the IR subsequently recruits IRS family proteins, which in turn recruit and activate PI3K; PI3K subsequently phosphorylates AKT-S473, a key phosphorylation site for insulin signaling transduction^34^. The present study indicated that BPA clearly inhibited IR pY1355, a tyrosine kinase phosphorylation site that was responsible for the transmission of upstream signaling pathways for insulin^4^. In further support of these results, we also determined that the serine phosphorylation of IRS-1, a hallmark feature of insulin resistance^35^, was substantially increased. Furthermore, BPA significantly inhibited the phosphorylation of AKT at serine 473 (an important upstream signaling component that regulates GSK-3β inactivation) and reduced phosphorylated GSK-3β at the serine 9 residue (which negatively reflects increased GSK-3β activity) and phosphorylated GSK-3α at the serine 21 residue (which negatively reflects increased GSK-3*α* activity) in SY5Y cells. To further identify the effects of BPA on the critical insulin signaling AKT, we explored the downstream signaling of AKT, mTOR and PP2A, which were shown closely associated with p-tau regulation^20, 36^. As we speculated, BPA exposure significantly decreased the phosphorylation of mTOR and methylation level of PP2A, suggesting the adverse effects of BPA on insulin signaling pathway. The increases in the activities of GSK-3α and β are responsible for APP generation and the hyper-phosphorylation of tau, respectively^37^; thus, these findings prompted us to infer that BPA exposure results in the enhancement of APP and p-tau expression. As speculated, the APP and p-tau expressions were substantially elevated after BPA exposure in SY5Y cells, and these effects were confirmed in another cell line, PC-12, which suggests that BPA affects APP and p-tau generation. Increased APP expression due to higher levels of the corresponding transcript would provide more substrate and an increased probability of aberrant cleavage, thereby generating Aβ, a neurotoxic polypeptide formed by the hydrolysis of APP via BACE-1^24^. We subsequently determined whether BPA exposure affected BACE-1 expression. In the current study, BPA significantly promoted BACE-1 expression, which may result in an increase in the expression of extracellular Aβ_1–42_. To further support the hydrolysis of APP mediated by BPA, the present work indicated that BPA obviously increased the C-terminal fragments of APP, including β-CTF and the α-CTF. These results underscore the importance of the IR/IRS-1/AKT/GSK-3α/APP axis disturbances in BPA-mediated AD-like neurotoxicity.

Neurofibrillary tangles (NFTs), formed by hyperphosphorylated tau protein misfolding, are another important feature of early AD development^38^. Over the past few years, interest in the association between impaired insulin signaling and hyperphosphorylated tau has increased^39^. However, to our knowledge, the underlying mechanisms of the BPA-mediated hyperphosphorylation of tau have not been fully elucidated. In the present study, we determined that BPA exposure substantially increased the p-tau expression in SY5Y cells, and this effect was confirmed in a PC-12 cell line in which multiple phosphorylation sites (Ser199, Ser396, Thr205, Ser214, and Ser404) were activated. These results may be attributed in part to the promotion of excessive GSK3β activation^40^. We suspect that BPA might affect microtubule assembly^41^; however, additional detailed studies are required to elucidate this phenomenon.

To dissect the detailed association between insulin signaling disturbances and APP and p-tau enhancement mediated by BPA, insulin and rosiglitazone were employed in this study. Rosiglitazone, a PPARγ agonist, has been reported to enhance the insulin sensitivity of cells and tissues, alleviate insulin resistance, and exert a protective effect on nerve cells^42^. Studies have indicated that after an intraperitoneal injection of rosiglitazone in an AD mouse model, spatial learning, memory and cognitive function were significantly improved^43^. Similarly, treatment with insulin, which has been shown to improve insulin signaling transduction in the brain, can help to improve AD patient scale score results^44^. In the present study, BPA-induced disturbances were substantially ameliorated by rosiglitazone treatment. Of note, the expression of pathological proteins regulated by BPA, including APP and p-tau, were substantially downregulated by rosiglitazone, which indicates the pivotal effects of insulin signaling pathways in BPA-mediated neural damage. These effects were also demonstrated in insulin-treated cells.

As an environmental estrogenic endocrine disruptor, BPA possesses a chemical structure similar to estradiol, and the estrogenic function of BPA was mainly characterized by investigations of the competitive binding of BPA to ERs^45^. Therefore, we investigated whether ERs participated in BPA-mediated pathological protein expression. The BPA-induced up-regulations of APP, BACE-1 and p-tau were clearly attenuated by the ICI182780 and G15 treatments, which thus emphasizes the role of ERs and GPR30 in this process.

**Figure 8:**
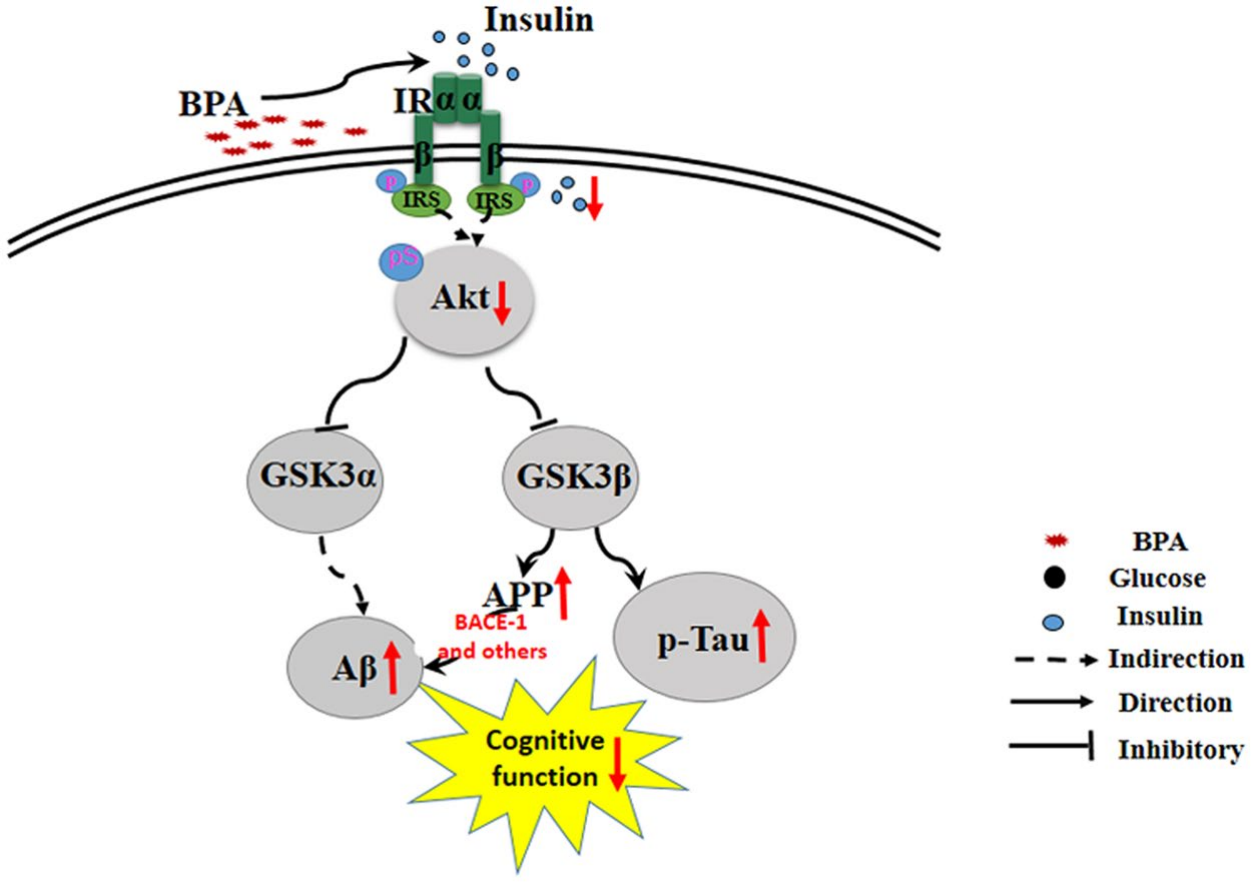
Hypothetical model of BPA-induced Alzheimer’s disease-like neurotoxicology. BPA disturbs the insulin signaling pathways by decreasing IR tyrosine phosphorylation and increasing IRS1 serine phosphorylation, which leads to reduced AKT phosphorylation. The inactivation of AKT subsequently results in the overactivation of GSK3α and GSK3β, two critical enzymes responsible for APP and p-tau formation. APP is subsequently hydrolyzed by BACE-1, which facilitates Aβ_1–42_ generation, and the increased Aβ_1–42_ expression and enhancement of p-tau result in an AD-like disease.

In summary, the present study unravels a novel mechanism of BPA-mediated pathological protein expression that involves the engagement of ERs, the disturbance of IR, IRS-1 and AKT signaling transduction and downstream GSK3α/β activation, and the increased expressions of APP, BACE-1, Aβ_1–42_, and p-tau, which ultimately culminate into an AD-like disease, as summarized in Fig. 8. Therefore, understanding the links between insulin signaling disturbances and pathological protein formation could lead to the development of therapeutic strategies that target BPA-induced AD-like disease.

## Materials and Methods

### Reagents

BPA (purity > 99%), RIPA lysis buffer, and protease inhibitor cocktail were obtained from Sigma (St. Louis, MO, USA). TRITC-conjugated goat anti-rabbit-IgG secondary antibody and a BCA Protein Assay Kit were purchased from Thermo Fisher (Rockford, IL, USA). Dulbecco’s Modified Eagle Medium (DMEM), fetal bovine serum (FBS), penicillin, and streptomycin were obtained from HyClone Laboratories Inc. (Legan, Utah, USA). The antibodies are listed in Table 1.

**Table 1:** Antibodies used in the study.

Antibody	Type	Specificity	Source
Insulin	Poly	Insulin	Proteintech (15848-1-AP)
p-IR	Poly	p-IR at Tyr 1355	Bioworld (BS4270)
IR	Mono	Total IR	Cell signaling (3025s)
p-IRS1	Poly	p-IRS1 at Ser 307	Affinity (AF3272)
p-IRS1	Mono	p-IRS1 at Tyr 896	Abcam (ab46800)
IRS1	Mono	Total IRS1	Abcam (ab131487)
p-AKT	Mono	p-AKT at Ser 473	Cell signaling (4060s)
AKT	Mono	Total AKT	Cell signaling (4691s)
p-GSK3β	Mono	p-GSK3β at Ser 9	Cell signaling (5558s)
GSK3β	Mono	Total GSK3β	Cell signaling (9315s)
p-GSK3α	Mono	p-GSK3α at Ser 21	Cell signaling (9316s)
GSK3α	Mono	Total GSK3α	Cell signaling (4337s)
APP	Mono	APP	Abcam (ab180140)
BACE-1	Mono	BACE-1	Cell signaling (5606s)
pT205-tau	Poly	p-tau at Thr205	Affinity (AF3150)
pS199-tau	Mono	p-tau at Ser 199	Abcam (ab81268)
pS396-tau	Mono	p-tau at Ser 396	Abcam (ab109390)
pS214-tau	Mono	p-tau at Ser 214	Abcam (ab170892)
pS404-tau	Poly	p-tau at Ser 404	Abcam (ab131338)
T-tau	Poly	Total Tau	Abcam (ab76128)
ML309-PP2Ac	Mono	methyl-PP2A	millipore (04-1479)
ps2481-mTOR	Poly	p-mTOR at Ser2481	Cell signaling (2974S)
mTOR	Mono	Total mTOR	Cell signaling (2983S)
APP	Poly	APP	Cell signaling (2452S)
APP	Poly	APP	Bioss (bs-0347R)
GAPDH	Mono	GAPDH	Affinity (T0004)

### Cell culture and Sample Treatment

Both SH-SY5Y cells (ATCC#ACS-4004) and PC-12 (ATCC# CRL-1721) cells were obtained from American Type Culture Collection (ATCC, Rockville, MD, USA). These cells were cultured in DMEM with high glucose supplemented with 10% FBS, penicillin (80 units/ml), and streptomycin sulfate (80 μg/ml) at 37 °C in a humidified atmosphere of 5% CO_2_. Cells were incubated with different concentrations of BPA at 0, 2, 20, 200, and 2000 nM/L.

### Intracelluar Ca^2+^ detection

Relative change in [Ca^2+^]_i_ was measured with fluo-4/AM (Thermo Scientific Rockford, IL, USA). Cells were grown on the glass bottom dish and incubated fluo-4/AM for 30 min at 37 °C in dark place. After been washed, cell fluorescence was detected by the confocal lasers canning microscope (Zeiss, Germany). Measurements were performed on 5–10 cells in one field of vision. Fluorescence images were collected at the excitation wavelength of 488 nm per15s. Raw intensity values were imported into Graphpad Prism5 software and normalized using the equation R = [(F − Frest)/Frest] × 100%, R represents normalized fluorescence intensity. F is fluorescence intensity at time t and Frest refers to the mean of at least 10 determinations of F taken during the control period.

### Mitochondrial membrane potential detection

Briefly, cells were incubated with JC-1 reagent (5 mg/mL, Beyotime, China) for 30 min at 37 °C. Subsequently, cells were collected, washed with PBS and then analyzed by flow cytometry (B D FACSAria^TM^ III).

### Measurement of the ATP levels

The content of ATP was determined by a commercial kit according to the instruction of the manufacturer (Beyotime, China).

### Reactive Oxygen Species detection

The DCFH-DA assay was used to detect the level of Reactive Oxygen Species (ROS). Briefly, the cells were loaded with DCFH-DA (Beyotime, China) at 100 μM, and were incubated for 30 min at 37 °C to allow cellular incorporation of this ester. Then the medium was changed to fresh medium and the oxidation of DCFH was measured by confocal laser scanning microscope (Zeiss, Germany).

### Western blot analysis

After BPA treatment, the cells were rapidly harvested using ice-cold RIPA lysis buffer (Sigma, St Louis, MO, USA) with protease inhibitor. The lysates were subsequently collected and centrifuged at 12,000 rpm for 10 min at 4 °C. Then, the protein concentrations in the supernatant fluid of the lysates were determined using Pierce BCA protein assay reagent (Thermo Scientific Rockford, IL, USA).

Equal quantities of protein (40 μg) in the lysates were resolved using 10% SDS–PAGE and subsequently transferred to 0.20-μm PVDF membranes (Millipore, USA). The membranes were subsequently blocked with a solution that contained 5% non-fat milk in TBST at room temperature for 2 h and incubated overnight with the primary antibodies at 4 °C on a shaker (Table 1). The next day, the primary antibodies were removed, and the membranes were washed three times and incubated with secondary antibodies for 2 h at room temperature. The membranes were again washed eight times with Tween 20/Tris-buffered saline (TBST). Antibody-binding bands were visualized with Chemiluminescent HRP Substrate (Millipore Corporation, Billerica, MA, USA) and normalized to GAPDH. All experiments were repeated at least three times.

### Enzyme-linked immunosorbent assay

To determine the levels of Aβ_1–42_ in the culture media, supernatant was collected and quantified using enzyme-linked immunosorbent assay (ELISA) kits (Newbioscience, China). Quantification of the ELISA results was performed using a microplate reader set to a test wavelength of 450 nm and corrected for absorbance at 540 nm, according to the manufacturer’s instructions. All experiments were repeated at least three times.

### Immunofluorescence analysis and confocal microscopy

For the immunofluorescence analysis, PC-12 cells were cultured on glass bottom dishes. After treatment with BPA, the cells were fixed for 30 min with 4% paraformaldehyde in PBS and followed with 0.2% Triton (−20 °C) for 5 min and three rinses in phosphate-buffered saline. The samples were incubated with 5% bovine serum albumin (BSA) in phosphate-buffered saline-Tween-20 (PBST) for 30 min and further incubated overnight at 4 °C with the primary antibodies rabbit polyclonal BACE-1 (1:100), Tau396 (1:50), and Tau404 (1:100). After washing with PBS, the dishes were further incubated with TRITC-conjugated goat anti-rabbit-IgG secondary antibody (1:100) for 2 h (37 °C). Finally, DAPI was added for nuclear staining. The samples were mounted and observed under a ZEISS confocal laser scanning microscope 700.

### Statistical analysis

All data were analyzed with SPSS 20.0 software. To test the statistical significance of the differences, one-way analysis of variance (ANOVA) and Dunnett multiple comparison procedures were used, as appropriate, for comparisons. Statistical significance was assumed at P < 0.05. A P value of > 0.10 was required to assess the homogeneity of variance across the groups.

## Electronic supplementary material

Supplementary figures

Supplementary video
