# Supplementary material for: Involvement of Insulin Signaling Disturbances in Bisphenol A-Induced Alzheimer’s Disease-like Neurotoxicity
Source: Sci Rep. 2017 Aug 8;7:7497. doi: 10.1038/s41598-017-07544-7 (PMC5548741; doi:10.1038/s41598-017-07544-7)
Supplement: Supplementary file 1 — Supplementary figures [file 41598_2017_7544_MOESM1_ESM.pdf]

Involvement of Insulin Signaling Disturbances in Bisphenol A-Induced Alzheimer's Disease-like Neurotoxicity

Tingwei Wang[1], Cuiwei Xie[1],Pengfei Yu[1] ,Fangfang Fang[1], Jingying Zhu[2], Jie Cheng[1], Aihua Gu[1], Jun Wang[1,*], Hang Xiao[1,*]

Figure:

Supplementary figure1.(S1)

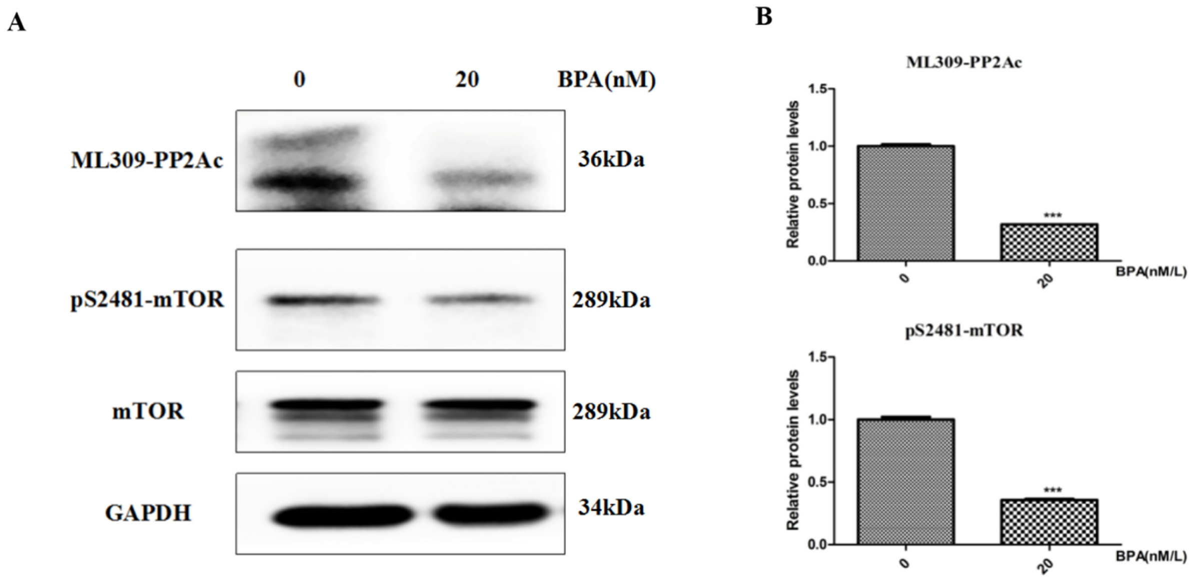

Supplementary figure2.(S2)

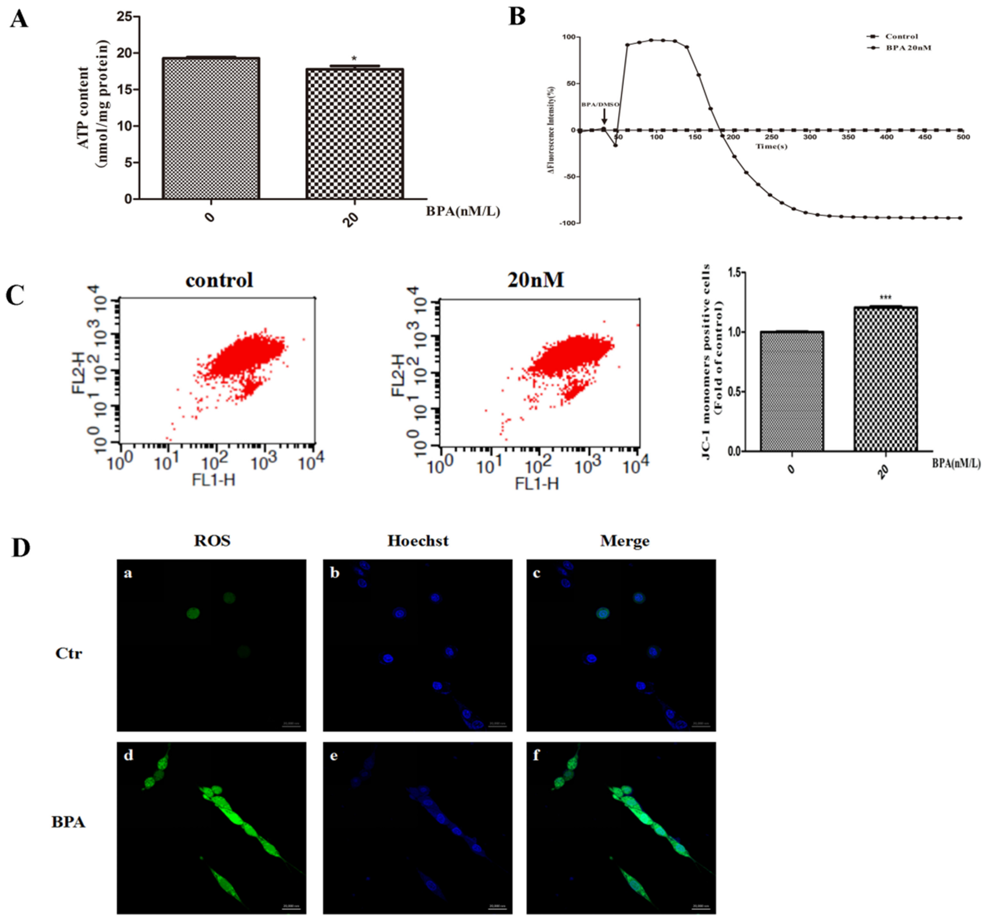

Supplementary figure3.(S3)

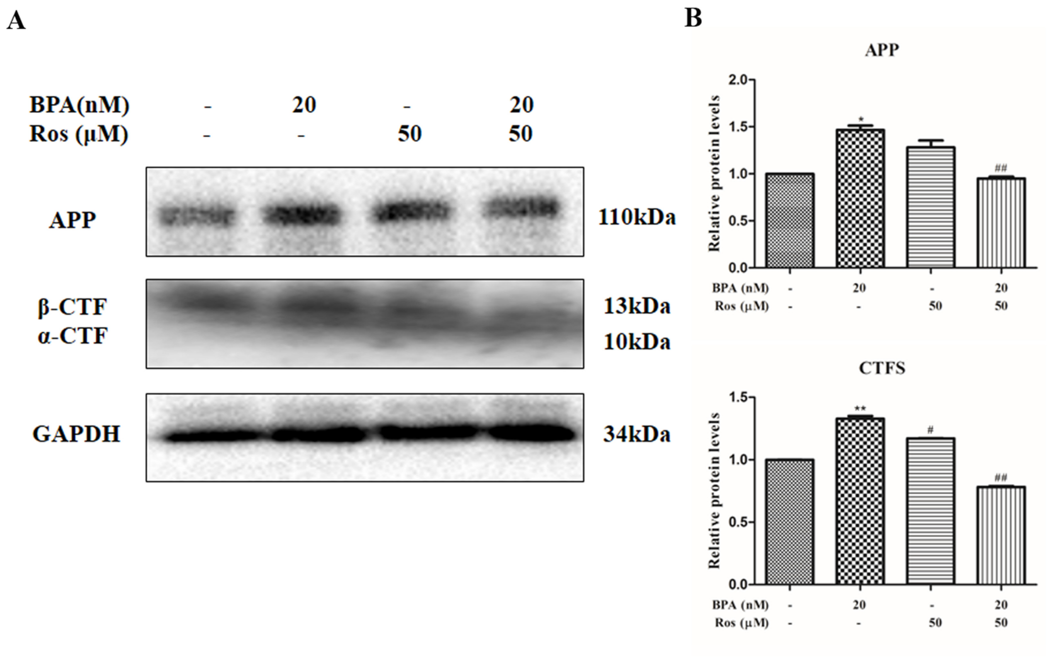

Supplementary figure4.(S4)

A

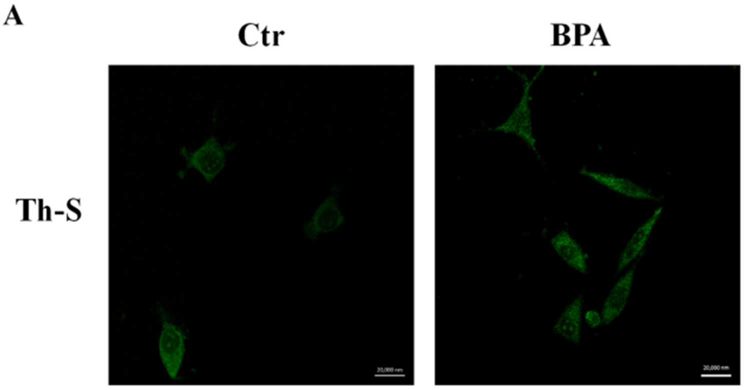

B

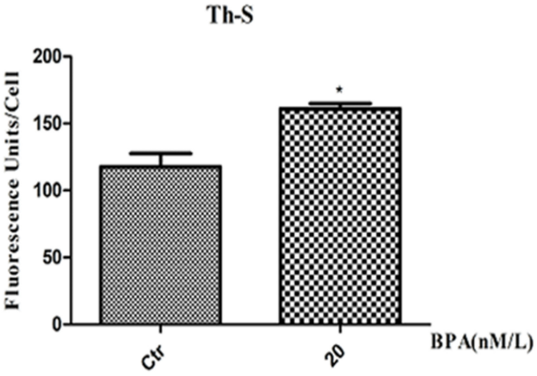

Supplementary figure5.(S5)

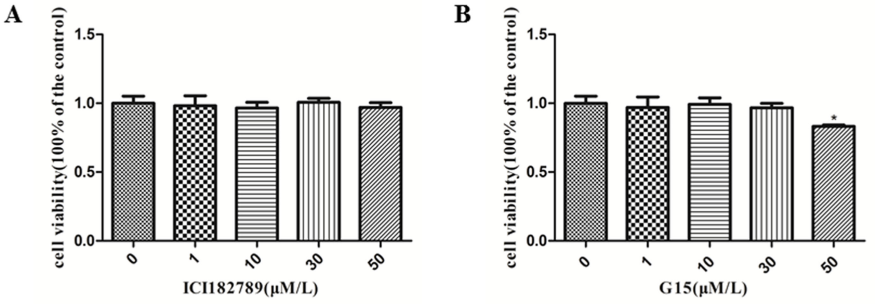

Supplementary figure6.(S6)

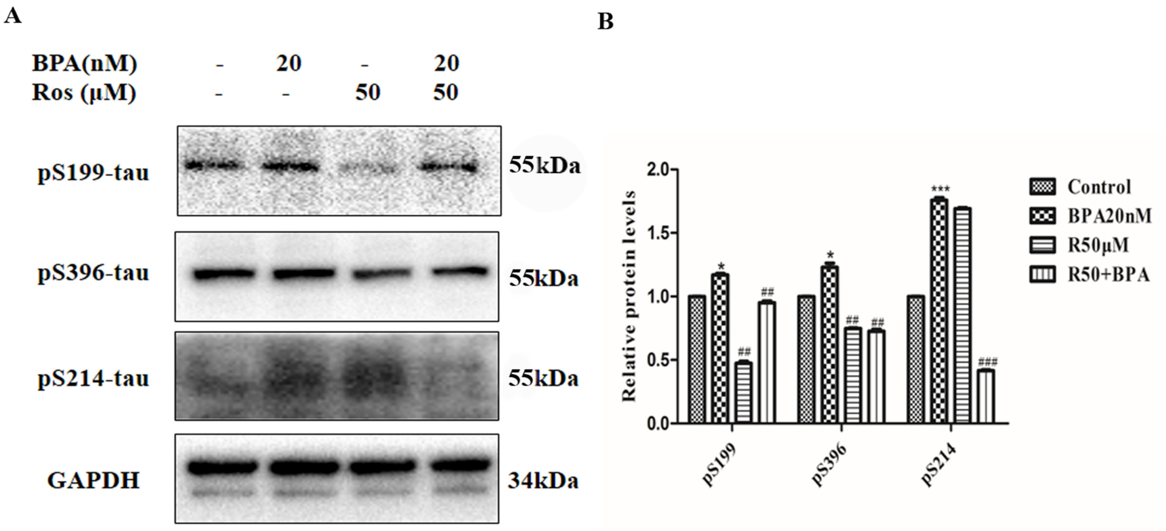

Supplementary Figure Legends

Fig.S1 Effects of BPA on the expression of mTOR phosphorylation and PP2A methylation. Phosphorylation of mTOR and Methylation level of PP2A were detected by western blot. GAPDH levels performed in parallel served as controls. Mean values ± SEM are representative of three independent isolations and three independent samples. Significant differences between the treatment groups and the control group were determined by one-way ANOVA and the Dunnett multiple comparison procedure (***$P < 0.001$ compared with the control group).

Fig.S2 Effects of BPA on $[Ca^{2+}]_i$ and mitochondrial associated dysfunction. (A)Effects of BPA on ATP generation. (B) Effects of BPA on intracellular $[Ca^{2+}]_i$. (C) Effects of BPA on mitochondrial membrane potential. (D) Effects of BPA on ROS level. Mean values ± SEM are representative of three independent isolations and three independent samples. Significant differences between the treatment groups and the control group were determined by one-way ANOVA and the Dunnett multiple comparison procedure (***$P < 0.001$ compared with the control group).

Fig.S3 Effects of BPA and rosiglitazone on α-CTF and β-CTF expression. Bands of

α-CTF and β-CTF were detected by western blot. GAPDH levels performed in parallel served as controls. Mean values ± SEM are representative of three independent isolations and three independent samples. Significant differences between the treatment groups and the control group were determined by one-way ANOVA and the Dunnett multiple comparison procedure (***P < 0.001 compared with the control group).

Fig.S4 Effects of BPA on pathological protein aggregation by staining with thioflavine-S. Fluorescence of thioflavin S on SY5Y cells were detected after treated with/without 20 nM/L BPA. Mean values ± SEMs are representative of three independent isolations and three independent samples. Significant differences between the treatment groups and the control group were determined via Student's T-Test.(*P < 0.05).

Fig.S5 Effects of ICI182789 and G15 on cell viability. The cells were pre-incubated with varied concentration of ICI182789 and G15 for 30 min, after 12 h, the cell viability was detected by CCK-8 assay. Mean values ± SEM are representative of three independent isolations and three independent samples. Significant differences between the treatment groups and the control group were determined by one-way ANOVA and the Dunnett multiple comparison procedure (*P < 0.05 compared with the control group).

Fig.S6 Effects of rosiglitazone on BPA mediated high phosphorylated tau in

SH-SY5Y cells. Effect of rosiglitazone on the expression of phosphorylated tau mediated by BPA in SY5Y cells. GAPDH levels were assessed in parallel and served as controls. Mean values ± SEMs are representative of three independent isolations and three independent samples. Significant differences between the treatment groups and the control group were determined via one-way ANOVA and the Dunnett multiple comparison procedure. (*$P < 0.05$, **$P < 0.01$ compared with the BPA single treatment group, #$P < 0.05$, ##$P < 0.01$).
