# Supplementary figures and images for: Involvement of Insulin Signaling Disturbances in Bisphenol A-Induced Alzheimer’s Disease-like Neurotoxicity
Source: Sci Rep. 2017 Aug 8;7:7497. doi: 10.1038/s41598-017-07544-7 (PMC5548741; doi:10.1038/s41598-017-07544-7)

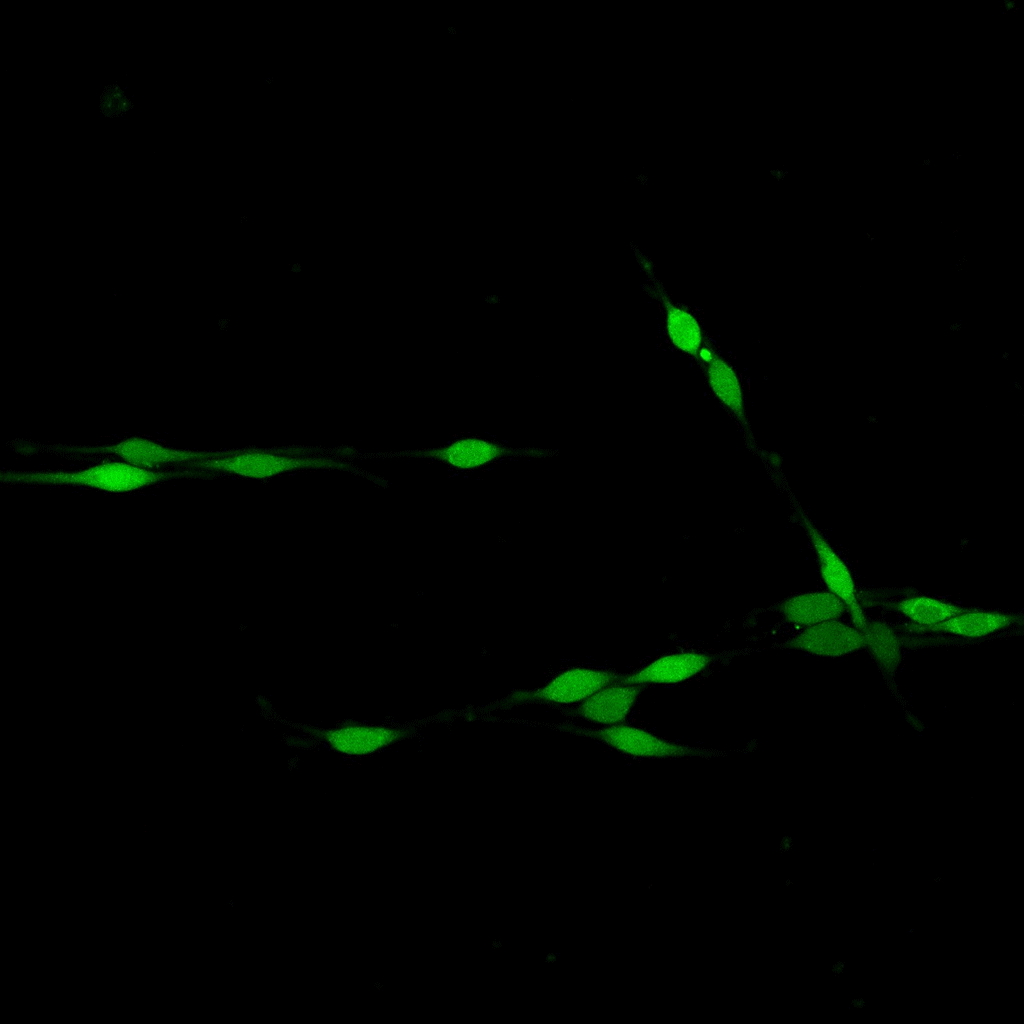

Supplement: Supplementary file 2 — Supplementary video [file 41598_2017_7544_MOESM2_ESM.gif]
